# Pair distribution function analysis of the reassembly step of the assembly-disassembly-organisation-reassembly (ADOR) process†

**DOI:** 10.1039/d2dt03389e

**Published:** 2022-11-23

**Authors:** Samantha E. Russell, Fanny N. Costa, Maria Diaz-Lopez, Russell E. Morris

**Affiliations:** EaStChem School of Chemistry, University of St Andrews Purdie Building St Andrews KY16 9ST UK rem1@st-andrews.ac.uk; Diamond Light Source Ltd, Diamond House Harwell Campus Didcot Oxfordshire OX11 0DE UK

## Abstract

An *in situ* pair distribution function study assessing the reassembly of three IM-12 (UTL) intermediate materials to the corresponding fully connected materials. A greater level of atomic change is observed at higher temperatures for the reassembly of the fully disconnected intermediate, IPC-1P, compared to the two partially connected intermediates of IPC-2P and IPC-6P.

## Abstract 2

Differences in the ADOR reassembly step were revealed by *in situ* pair distribution function analysis.

## Graphical abstract

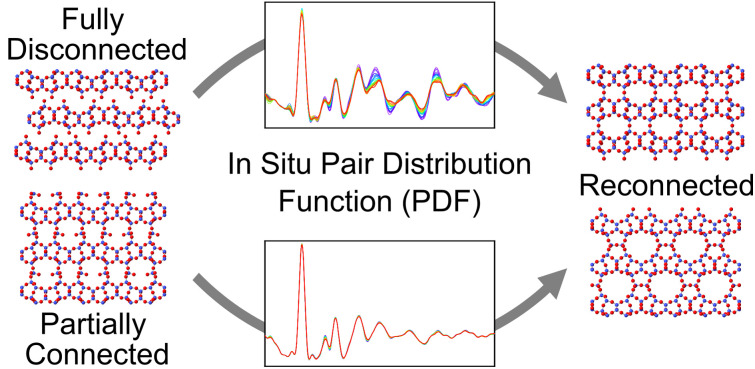

The assembly-disassembly-organisation-reassembly (ADOR) process is a synthesis method that allows the formation of zeolites that are unobtainable by standard hydrothermal conditions.^1–5^ The process consists of several steps: the assembly of a parent zeolite (A); the disassembly into an intermediate state (D); an organisation or rearrangement step of the intermediates (O) and a final reassembly step to form a new, fully connected material (R). By nature of the bond-breaking and rearrangement of the ADOR process, there is a loss of long range order in the structures as the reaction proceeds through the intermediates, before being restored after the reassembly step.^6,7^ Owing to this disorder, pair distribution function (PDF) analysis is one of the most useful tools to probe the mechanism at an atomic level.^8,9^ PDF data yields information on the local interatomic distances in a material, and can be particularly useful to look at changes in these distances during reactions as this gives vital information on the mechanisms at play. While PDF has previously been used to study the disassembly and organisation steps of the ADOR process,^10–12^ here we present for the first time a PDF study on the reassembly. The reassembly is a crucial step that forms the final fully connected zeolite that, due to the beauty of the ADOR process, differs from the original starting zeolite.

In this study we focus on the reassembly of three key intermediates from the disassembly of the parent zeolite IM-12 (UTL); they are IPC-1P to IPC-4, IPC-2P to IPC-2 and IPC-6P to IPC-6. The final structures differ by the linkages between the highly siliceous layers, where IPC-4 is connected through direct oxygen linkages, IPC-2 by single four rings (s4r) and IPC-6 a 50 : 50 combination of direct oxygen linkages and s4r, as shown in Fig. 1. The reassembly occurs by calcination of the intermediate materials, where the free silanol groups condense to form fully connected linkages between the zeolite layers. To probe the reassembly process in real time, we used *in situ* PDF analysis to monitor the atomic changes of the intermediate materials with increasing temperature as the materials calcined to the fully connected structures.

**Fig. 1:**
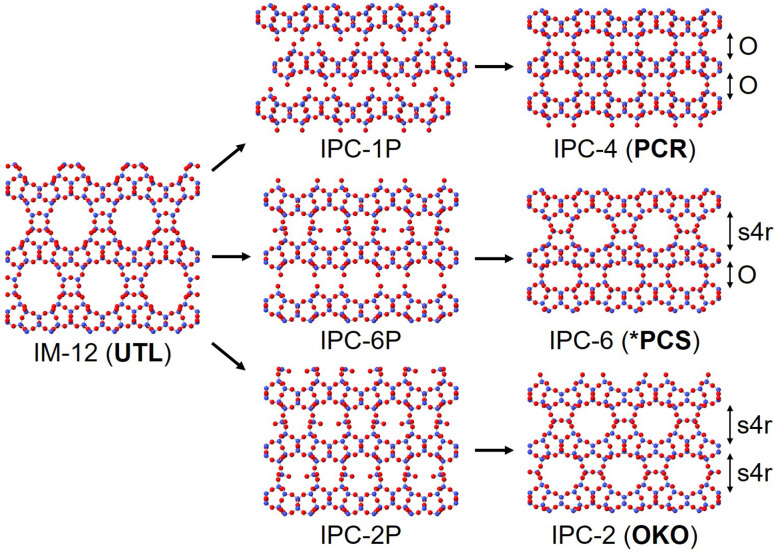
The structures of the three intermediate materials; IPC-1P, IPC-6P and IPC-2P, that are produced from different hydrolysis conditions of IM-12 (UTL). The final fully connected structures IPC-4 (PCR), IPC-6 (*PCS) and IPC-2 (OKO) are produced upon calcination. The three final materials have different layer linkages, with “O” representing direct oxygen linkages and “s4r” representing single-4-rings.

The initial IM-12 (UTL) parent zeolite and the desired intermediates of IPC-1P, IPC-2P and IPC-6P were synthesised as described in the ESI.† In-house powder X-ray diffraction (PXRD) was used to confirm the successful synthesis of all materials. Calcination of part of the intermediate samples, followed by subsequent PXRD, was also performed to confirm that the expected final product was obtained from each intermediate. Since the interlayer distance changes dependant on the connections present, monitoring the position of the *d*_200_ peak, which corresponds to the interlayer distance, is a key way to identify the products obtained.^13,14^ Generally, the interlayer distances of the fully connected materials are much more reliable than the interlayer distances of the unconnected or partially connected intermediates, hence why PXRD of the calcined materials was collected to confirm the intermediate materials.

X-ray total scattering measurements were collected at beamline I15-1 at Diamond Light Source, using an X-ray energy of 76.7 keV (*λ* = 0.161669 Å). The hydrolysed intermediates were packed in capillaries and topped with glass wool to hold the sample in place while simultaneously allowing water produced from the silanol condensations to be removed throughout the calcination. The IPC-1P calcination conditions ran from 30 °C to 230 °C in 50 °C steps, followed by 230 °C to 330 °C in 25 °C steps, then 330 °C to 550 °C in 10 °C steps and finished with two final measurements taken at 570 °C and 575 °C. Shorter data collection strategies were used for both the IPC-2P and IPC-6P calcinations due to time constraints. These calcination conditions ran from 30 °C to 180 °C in 50 °C steps, followed by 180 °C to 420 °C in 20 °C steps and final measurements at 450 °C, 500 °C, 550 °C and 570 °C. The 2D to 1D diffraction patterns were converted using DAWN and the PDFs were extracted using GudrunX.^15,16^

Two different heat sources were necessary to cover the full temperature range. Initial “low temperature” measurements from 30 °C–180 °C were collected using an Oxford Instruments Cryojet5, followed by the remaining “high temperature” measurements from 180 °C–570/575 °C using a FMB Oxford hot air gas blower. Intensity differences between the two sets of measurements have led to the low and high temperature data sets being treated separately. Further to this, there was a loss of beam during the data collections at 400 °C and 420 °C for IPC-2P, therefore these measurements are not presented.

The Bragg data, Fig. 2, was initially assessed to ensure the calcinations had successfully gone to completion under the *in situ* conditions. The Bragg data showed the *d*_200_ peak shifts that would be expected when calcining these intermediate materials,^13^ indicating successful reassembly to the final products.

**Fig. 2:**
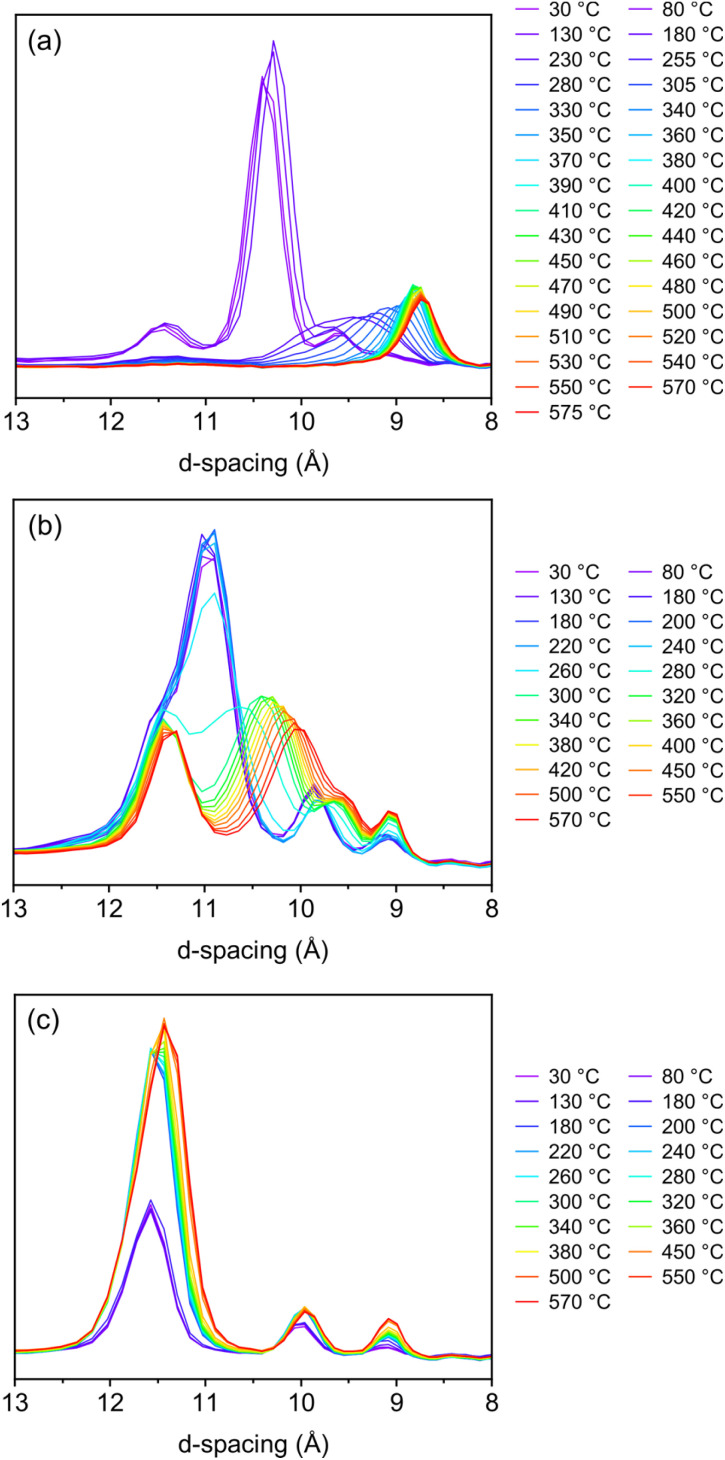
The Bragg data of the *d*_200_ peak for the *in situ* calcination of (a) IPC-1P to IPC-4, (b) IPC-6P to IPC-6 and (c) IPC-2P to IPC-2. All peak shifts are in line with what would be expected for the calcination of these materials. The duplication of the 180 °C temperature is due to one data collection obtained with low temperature measurements and one obtained with high temperature measurements.

Firstly, considering the *in situ* low temperature PDF data, Fig. S1,† in this 30–180 °C range we witness silicon rearrangement taking place in the interlayer regions. This is something that has been observed under aqueous conditions at 100 °C in previous *in situ* PDF work.^12^ In this instance, however, we can see this rearrangement occurring in the absence of any liquid, just an elevated temperature. The fact that the IPC-2P material contains highly siliceous, partially connected interlayer regions, explains why we witness PDF changes indicative of this silicon lability at these lower temperatures. Contrastingly, we see minimal changes to the PDF data for IPC-1P, the intermediate which contains no interlayer silicon due to total d4r removal without any further silicon re-intercalation during the disassembly step. The IPC-6P low temperature changes reside somewhere between these materials, with slightly more peak changes than IPC-1P but far less than the IPC-2P, indicating a small degree of silicon rearrangement occurring.

Also in this low temperature range, we would expect to see any material changes occurring due to water loss from the pores or surrounding system. Again, due to the partially connected interlayer region of IPC-2P, it is likely there may be more of an impact on the material as water is removed from these porous regions. This further adds to the understanding of why we observe more significant material changes for IPC-2P, by PDF, than IPC-1P or IPC-6P.

Considering the high temperature *in situ* PDFs, we observe a much higher degree of movement from the IPC-1P calcination compared to that of IPC-2P and IPC-6P, as seen in Fig. S2.† A number of the IPC-1P peaks show noticeable changes to the intensities and shapes as the calcination proceeds, this is in contrast to IPC-2P and IPC-6P which show minimal changes, except for a couple of select peaks. Notably, this indicates that a different process is occurring in this high temperature range of 180–570/575 °C compared to the low temperature range. Here we witness the condensation of the silanol groups and the structural organisation that is required to achieve this. If we consider that IPC-1P is the only fully disconnected structure, as displayed in Fig. 1, it would be reasonable to expect to see the most changes in the PDF as the structure organises and reconnects with increasing temperature. IPC-2P and IPC-6P, on the other hand, are both partially reconnected structures, therefore the layers are essentially already in the correct position for reassembly and hence we see less movement.

A second observation is the distance range that we observe peaks in the PDF, indicating the degree of long-range order that is present. The IPC-1P sample shows peaks out to much greater distances than the IPC-2P and IPC-6P samples, indicating the harsher acidic conditions and longer hydrolysis times have had an impact on the long range structure.

Difference plots were calculated for the high temperature PDF data by subtracting the first data set from the last data set to determine the peaks undergoing the most significant changes throughout the calcination. These plots were then compared to the corresponding partial PDFs to, where possible, assign the differences to a specific atomic pair, something that becomes much more difficult at higher distances due to significant overlap.

First, assessing the difference and partial PDFs from the IPC-1P to IPC-4 calcination, Fig. 3a, there is a particularly interesting peak that grows throughout the calcination at 5.6 Å. The reason for this interest is the fact it is a potentially new atomic distance that is formed during the calcination, with no peak present in that position with the first data set. Upon inspection of the fully calcined IPC-4 crystal structure, there is a distance around 5.6 Å that is present between neighbouring bridging oxygen atoms that connect the layers, Fig. S3a.† This suggests that as the silanol groups condense, the resulting bridging oxygen atoms are increasing in rigidity and therefore providing an average interatomic distance that was not observed when they were present as free silanol groups at the edge of the layered material.

**Fig. 3:**
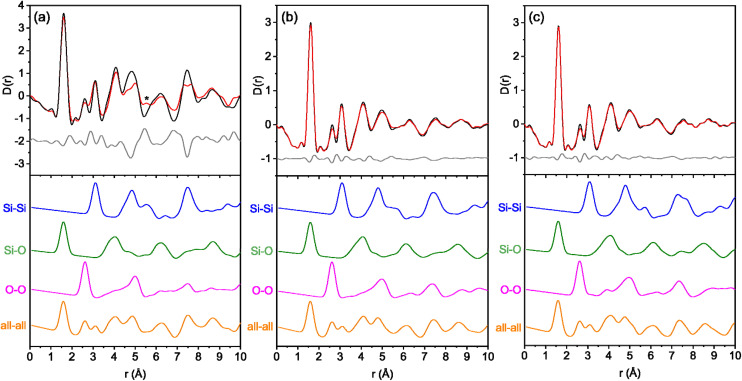
The difference PDF and partial PDFs of (a) IPC-1P to IPC-4, (b) IPC-6P to IPC-6 and (c) IPC-2P to IPC-2. The top graphs show the first (black) and last (red) high temperature measurements and the calculated difference between them (grey). The asterisk highlights the peak growth at 5.6 Å for the IPC-1P to IPC-4 reassembly. The bottom graphs show the calculated partial PDFs of (a) IPC-4, (b) IPC-6 and (c) IPC-2 showing all atom pairs (orange), O–O pairs (pink), Si–O pairs (green) and Si–Si pairs (blue).

Alongside this new peak, two peaks noticeably decrease in intensity as the IPC-1P calcination proceeds. They are at a distance of 4.9 Å and 7.6 Å, which according to the partial PDFs correspond to contributions from Si–Si and/or O–O for both peaks. Observing the bond distances in the IPC-4 crystal structure, both of these O–O distances are partly represented by atom pairs that involve the bridging oxygen atom between the layers. The shorter 4.9 Å distance is present between the bridging oxygen and an oxygen atom in the middle of the layer, Fig. S3b,† while the 7.6 Å distance includes an oxygen atom at the edge of the layer, Fig. S3c.† Therefore, this gradual decrease in peak intensity likely represents the reduction in the number of oxygen atoms present as the silanol condensations occur.

The difference and partial PDF data for the IPC-2P to IPC-2 and IPC-6P to IPC-6 calcinations indicate that they both follow the same mechanism, with both PDFs showing extremely similar changes as the calcinations proceed (Fig. 3b and c). Since IPC-2P and IPC-6P are both partially connected structures, with 50% of the same s4r interlayer linkages, this may explain why we observe such similar reassembly mechanisms. One of the main peaks that changes over time is at 2.6 Å, which decreases as the reassembly proceeds. This peak corresponds to the primary O–O interatomic distance and reduces over time as the silanol groups condense, removing oxygen-atoms in the condensation and therefore reducing the number of O–O distances present. Besides this peak, there are very few other changes observed in the PDFs, indicating minimal movement from either material throughout the calcination.

Considering the temperatures that these changes are occurring, we see from the PDFs for IPC-1P to IPC-4 that the majority of the atomic changes take place between 300–500 °C. However, if we look at the changes in the Bragg data for the same IPC-1P calcination, we see this temperature range is lower, around 200–400 °C. This highlights that even once the layers are correctly arranged, as observed by the position of the *d*_200_ peak, there are still atomic-level changes occurring as bonds are formed to fully connect the layers, as observed in the *in situ* PDF data. This shows the additional information that can be gained with PDF and highlights the benefit of using PDF analysis to probe the diffuse scattering that is present in the ADOR process. Owing to such subtle changes in the IPC-2P and IPC-6P PDFs, it is harder to identify specific temperatures. For the IPC-6P Bragg data however, we do very clearly see a change commencing at 230 °C where we begin to observe the presence of two distinct layer distances.

Overall, the PDF data shows the differences between the IPC-1P reassembly compared to that of IPC-2P and IPC-6P. The fully disconnected IPC-1P intermediate shows a much higher degree of movement at higher temperatures in comparison to the two partially connected IPC-2P and IPC-6P intermediates. This indicates that a greater level of layer organisation is required to form IPC-4 than that for IPC-6 or IPC-2 formation. In all cases we observe the expected decrease in O–O atom pairs as the silanol groups condense at 200 °C+, but additionally for the IPC-1P to IPC-4 calcination, we observe clear framework changes as the material aligns and reassembles.

## Notes

The research data supporting this publication can be accessed at https://doi.org/10.17630/80b4c93c-aac7-4016-912a-b731a90e66f6.^17^

## Conflicts of interest

There are no conflicts to declare.

## Supplementary Material

DT-051-D2DT03389E-s001
