# Supplementary material for: Pair distribution function analysis of the reassembly step of the assembly-disassembly-organisation-reassembly (ADOR) process
Source: Dalton Trans. 2022 Nov 23;51(47):17947–51. doi: 10.1039/d2dt03389e (PMC9724949; doi:10.1039/d2dt03389e)
Supplement: DT-051-D2DT03389E-s001 [file DT-051-D2DT03389E-s001.pdf]

Electronic Supplementary Material (ESI) for Dalton Transactions.
This journal is © The Royal Society of Chemistry 2022

Pair distribution function analysis of the reassembly step of the assembly-disassembly-organisation-reassembly (ADOR) process

Samantha E. Russell, Fanny N. Costa, Maria Diaz-Lopez and Russell E. Morris

Synthesis Conditions

The parent zeolite IM-12 (**UTL**) was synthesised following the literature procedure.[1] The material was calcined at 575 °C for 6 h with a heating ramp of 2 °C/min to remove the organic structure directing agent (SDA). A range of conditions were used to hydrolyse the calcined IM-12 (**UTL**) in an attempt to obtain the desired intermediate materials of IPC-1P, IPC-2P and IPC-6P. IPC-1P was formed by hydrolysing the UTL in water at 95 °C for 4 h, IPC-2P with 1.5 M hydrochloric acid at 95 °C for 20 h and IPC-6P with 2 M hydrochloric acid at 95 °C for 9 h.

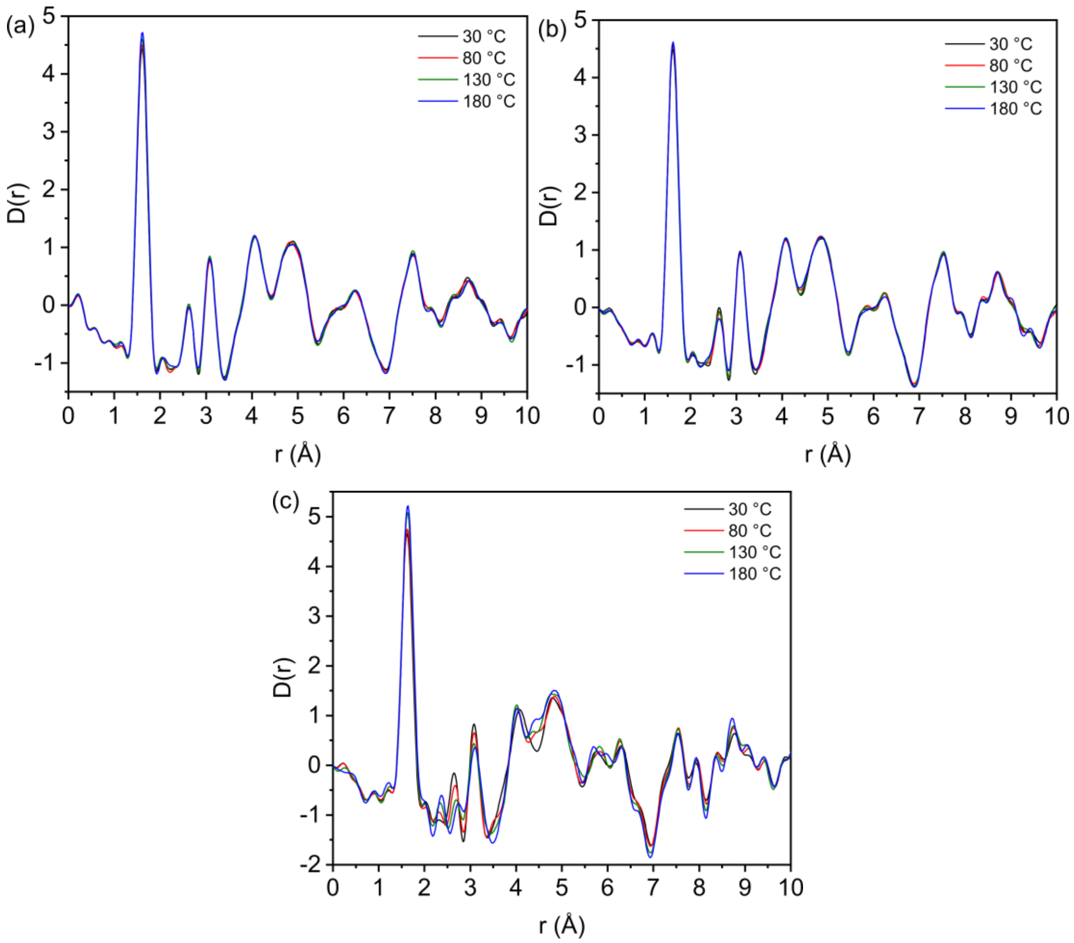

Figure S1. The low temperature 30 – 180 °C PDF data for the 0 – 10 Å region for (a) IPC-1P, (b) IPC-6P and (c) IPC-2P.

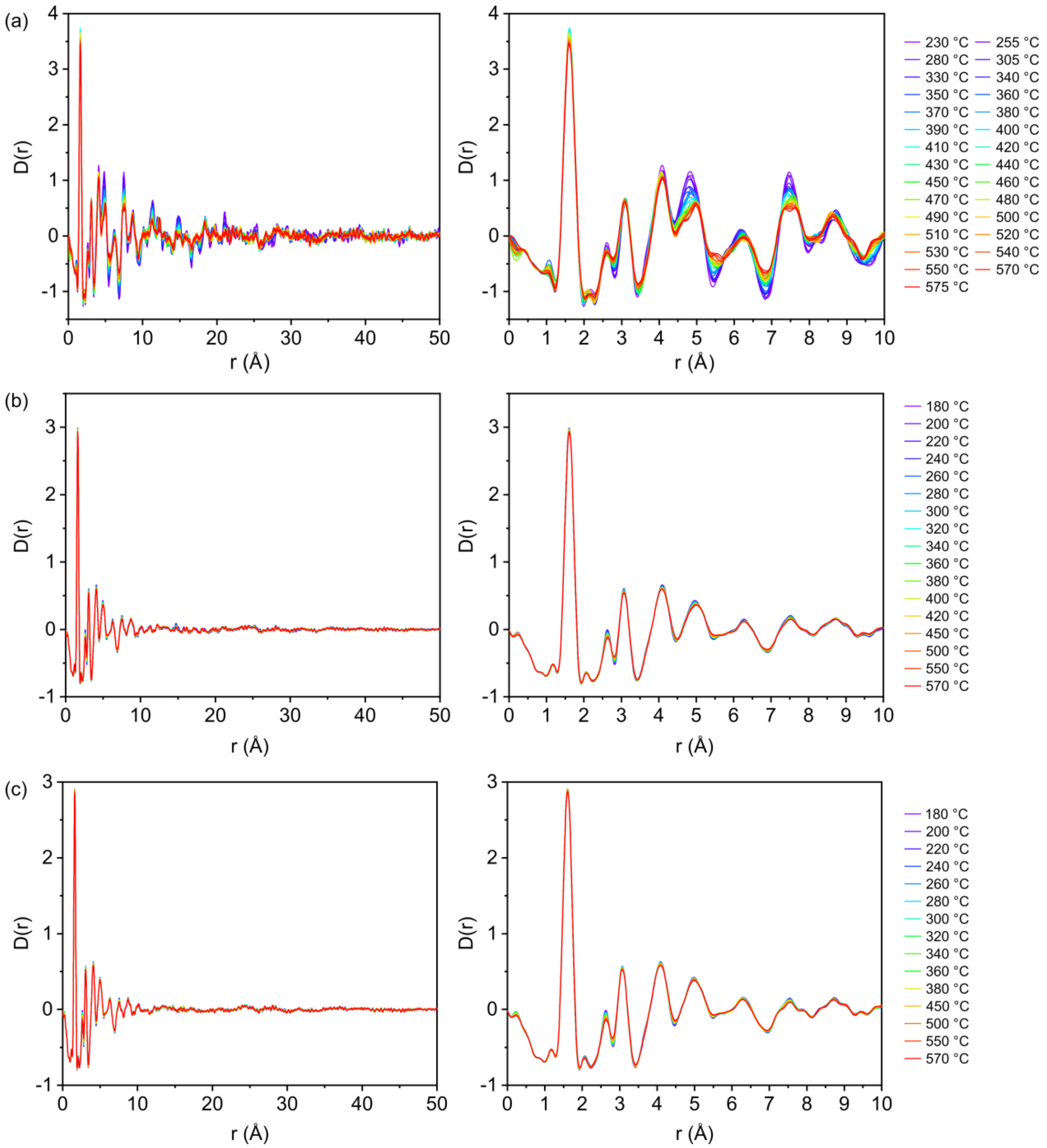

Figure S2. The full range of high temperature PDF data between 0 – 50 Å (left) and the zoomed region of 0 – 10 Å (right) for (a) IPC-1P, (b) IPC-6P and (c) IPC-2P.

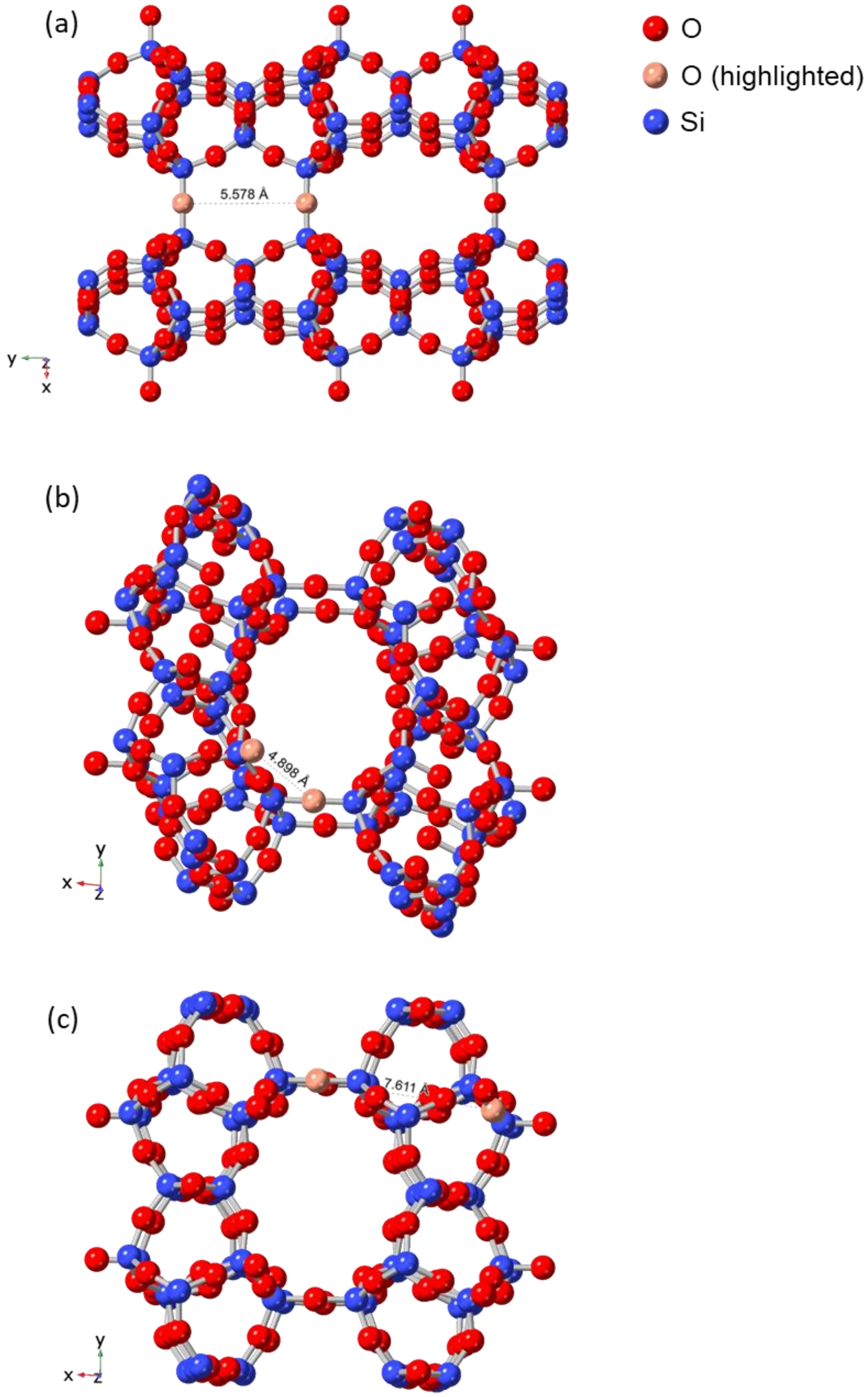

Figure S3: The IPC-4 crystal structure with the highlighted interatomic distances that were observed in the PDF data, where red are oxygen atoms, light pink are the highlighted oxygen atoms and blue are silicon atoms. (a) The distance of 5.6 Å between the two bridging oxygen atoms, (b) the 4.9 Å distance from the bridging oxygen to an "inner-edge" layer oxygen and (c) the 7.6 Å distance from the bridging oxygen to an "outer-edge" layer oxygen.

References

1. S. E. Henkelis, S. A. Morris, M. Mazur, P. S. Wheatley, L. N. McHugh and R. E. Morris, *J. Mater. Chem. A*, 2018, **6**, 17011–17018.
